# Eleven Years of Experience in the Treatment of Aortoiliac Aneurysm with the E-Liac Stent-Graft System

**DOI:** 10.3390/jcm14228203

**Published:** 2025-11-19

**Authors:** Enrique M. San Norberto, Álvaro Revilla, José Antonio Brizuela, Isabel del Blanco, Sergio Fernández-Bello, James H. Taylor

**Affiliations:** 1 Department of Vascular Surgery, Valladolid University Hospital, Av. Ramón y Cajal 3, 47005 Valladolid, Spainjabrizuela@saludcastillayleon.es (J.A.B.);; 2 Vascular Surgery Department, Croydon University Hospital, London CR7 7YE, UK

**Keywords:** aortic aneurysm, iliac aneurysm, endovascular procedures, endovascular aneurysm repair, iliac artery

## Abstract

**Background**: This study examines the use of the E-liac stent-graft system for the endovascular treatment of aortoiliac or isolated common iliac aneurysms. **Methods**: Consecutive patients between January 2014 and December 2024 were included. Data on patient characteristics, clinical presentation, lesion features, procedural aspects, and follow-up outcomes were collected and examined. Complications during the perioperative period and subsequent reinterventions were also documented. **Results**: A total of 81 patients met the inclusion criteria (65 men, 80.2%, median age 71.2 ± 11.43 (range 61–86 years). Technical success was achieved in all cases. A total of 97 internal iliac arteries were revascularized; in 54 cases (66.7%), the endovascular technique was EVAR + unilateral iliac branch device (IBD), in 16 cases (19.8%), it was EVAR + bilateral IBDs, and unilateral isolated IBD was conducted in 11 (13.6%) patients. The median patient follow-up time was 64.7 months (range 1–120). During follow-up, the mortality rate was 22.2%, with an iliac branch patency of 90.1%. Buttock claudication was observed in five (6.2%) patients and nerve ischemia in one (1.2%). One type I endoleak (1.2%) occurred following endovascular treatment of an isolated common iliac artery aneurysm, and three type II endoleaks (3.7%) were observed; none of them were associated with aneurysm sac enlargement. Three type III endoleaks (3.7%) occurred due to disconnection of the iliac branch from the extension of the concomitant EVAR. **Conclusions**: This long-term study, with 11 years of follow-up, reports outcomes with the E-liac stent-graft for the treatment of aorto-iliac or iliac aneurysms and demonstrates that it can be safely applied with low mortality and reintervention rates, and high patency rates.

## 1. Introduction

The endovascular repair of aortoiliac aneurysms presents a significant challenge, particularly when the aneurysmal disease extends to the common iliac artery, threatening the integrity of the internal iliac artery (IIA). Conventional endovascular aneurysm repair (EVAR) often necessitates intentional embolization or occlusion of the IIA to achieve adequate distal seal. However, this practice is associated with a risk of pelvic ischemia, leading to complications, such as buttock claudication, erectile dysfunction, and in rare cases, colonic ischemia or spinal cord injury. To mitigate these risks and preserve pelvic perfusion, iliac branch devices (IBDs) have emerged as a viable and increasingly adopted endovascular solution [1].

IBDs are modular stent grafts designed to maintain antegrade blood flow into the IIA while effectively excluding the aneurysm. The use of these devices has expanded the anatomical suitability for EVAR, allowing for a fully endovascular approach in complex aorto-iliac pathologies. The technical success rate with modern IBDs is high, ranging from 85% to 100% in published series, with an improving trend attributed to both device refinement and increased operator experience. The primary clinical benefit is the substantial reduction in pelvic ischemic complications, particularly buttock claudication, which has been reported at rates as low as 0–4% with IBDs compared to 16–55% with IIA occlusion [1,2].

The use of IBDs is growing in acceptance. In accordance with this trend, a comprehensive evaluation of IBDs use regarding their strengths but also their limitations, and especially the identification of the recommended indications, remains of high scientific interest. Three off-the-shelf IBDs are available in Europe: the ZBIS (Cook Medical, Bloomington, USA), the IBE (Gore, Flagstaff, USA), and the E-liac (Artivion, Hechingen, Germany), although other devices have been described worldwide (such as the Braile or Lifetech IBDs) [3]. Currently, the main anatomical and clinical indications for iliac branched devices include aortic aneurysms exceeding 25 mm extending to the iliac bifurcation, coexisting aneurysmal involvement of the hypogastric artery, contralateral occlusion of the hypogastric artery, and young patients with an active lifestyle who stand to benefit from techniques maintaining perfusion of the hypogastric artery; however, we believe that iliac preservation should be the standard of care in patients with suitable anatomy. While bilateral preservation with IBDs is technically feasible and has shown favorable results, the benefits must be weighed against the increased device costs, radiation dose, and procedure time. The major limitation for their use remains anatomic requirements. Certain anatomic restrictions must be considered before implantation: length and diameter of the external iliac artery, excessive iliac tortuosity, calcification, angulation between the common and the internal iliac arteries, intraluminal thrombus, and iliac stenosis [1,2].

Among the commercially available IBDs, the E-liac^®^ Stent Graft System (Artivion/Jotec) is a notable example. This system is specifically indicated for the treatment of aorto-iliac and isolated iliac aneurysms, offering a dedicated endovascular solution for preserving the hypogastric artery. The E-liac^®^ device features a pre-cannulated side branch, designed to facilitate rapid and precise deployment of a bridging stent into the IIA [4]. Clinical data for the E-liac^®^ system demonstrate favorable outcomes, including high primary patency rates for both the external and internal iliac arteries and low reintervention rates. A single-center study reported a technical success rate of 95% and a mid-term IIA patency of 97.6% [5]. Another multicenter study confirmed high technical success and a low incidence of buttock claudication, validating the E-liac^®^ system as a safe and effective option. While IBDs such as the E-liac^®^ system offer significant advantages, their use requires careful patient selection and a learning curve for operators. Continuous postoperative surveillance is also essential to monitor for potential complications such as endoleaks or device migration, which, though infrequent, necessitate ongoing follow-up to ensure long-term clinical success [5,6,7,8].

The aims of the current study were to report the short- and medium-term outcomes of patients with aorto-iliac or isolated common iliac aneurysms treated with the E-liac^®^ Stent Graft System in a real-world setting.

## 2. Materials and Methods

### 2.1. Study Design

This retrospective cohort study included consecutive electively treated patients who underwent endovascular treatment of aortoiliac or isolated common iliac artery aneurysms with iliac branch devices between January 2014 and December 2024 at Valladolid University Hospital, a Spanish tertiary center. For this analysis, only those treated with the E-liac^®^ Stent Graft System were included in the study cohort; contemporaneous patients managed with other IBD technologies or alternative endovascular approaches, including the bell-bottom technique, internal iliac artery coil embolization/occlusion, and parallel graft configurations (chimney/sandwich techniques), were excluded. The study, which received no financial support from industry, was performed in accordance with the Declaration of Helsinki and approved by the institutional review board. In accordance with institutional and local regulatory policies, this retrospective review of de-identified procedural and follow-up data was exempt from informed consent requirements. The study design and this manuscript were adapted to the STROBE guidelines for observational studies (see Supplementary Table S1).

Clinical indications included management of common iliac artery (CIA) aneurysms, treatment of secondary type Ib endoleaks caused by post-EVAR dilatation of the distal CIA fixation site, or use as an initial alternative approach to bell-bottom deployment (CIA > 20 mm). Inclusion criteria were patients with aorto-iliac or isolated common iliac aneurysms treated with endovascular exclusion via elective implantation of an E-liac^®^ Stent Graft System. Patients presenting with symptomatic or ruptured iliac aneurysms, as well as those who underwent open surgical reconstruction, were excluded. Additional exclusion criteria included patients who could not receive antiplatelet or anticoagulation therapies or had very limited life expectancy (defined as individuals with a terminal illness expected to live for one year or less). The anatomical complexity and vascular morphology of aneurysms included in the study are presented in Table 1.

The decision to implant an E-liac stent-graft or another IBD (such as the Gore Excluder^®^ iliac branch endoprosthesis or Zenith^®^ iliac branch device) was left to the operator’s discretion. All operators were qualified consultant vascular surgeons. Other treatment modalities for aortoiliac or isolated common iliac aneurysms included standard EVAR, bell-bottom technique, hypogastric coil embolization/occlusion, or chimney/sandwich techniques. All patients treated with the E-liac IBD since its commercial launch in 2014 were included.

### 2.2. Patients and Follow-Up

**Figure 1:**
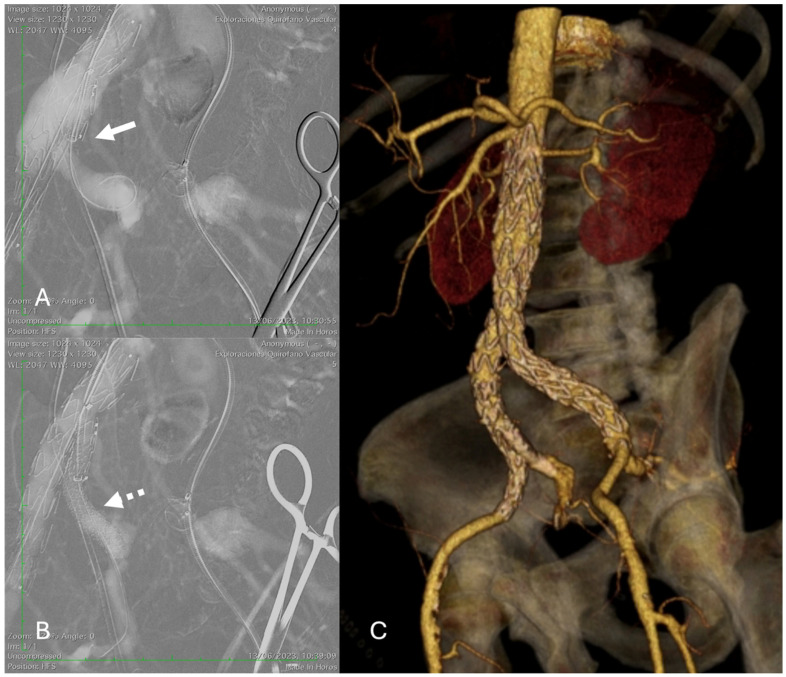
E-tegra (Artivion/Jotec) and E-liac stent-graft system (Artivion/Jotec) implantation in a patient with an infrarenal AAA and right common iliac artery aneurysm. (**A**) Digital subtraction CO_2_ angiography shows the main limb of the device deployed with the markers facing the iliac stump (white arrow) and cannulation of the stump and the right internal iliac artery. (**B**) The bridging stent (E-ventus 9 × 57 mm, Artivion/Jotec) was placed into the right internal iliac artery (dotted white arrow). (**C**) At 12-month follow-up, 3D reconstruction CT angiography shows successful exclusion of the aneurysms.

Demographics, clinical status, medical history, and procedural aspects were recorded. IBD implantation was performed using the technique previously described by Mylonas et al. [9]. A dual antiplatelet regimen of aspirin (100 mg daily) and clopidogrel (75 mg daily) was initiated after the procedure for at least 4 weeks, followed by lifelong continuation of single-agent antiplatelet therapy (Figure 1).

Final angiographic assessment using digital subtraction angiography (DSA) was performed after each intervention to document the implanted stent site and evaluate downstream vessel patency for evidence of endoleaks or other complications (such as external or internal iliac dissection, thrombosis, or embolism). However, in patients at high risk of complications from iodinated contrast medium, such as those with a history of allergic reaction or impaired renal function (eGFR < 30 mL/min/1.73 m^2^), CO_2_-DSA was used.

All patients were followed in a postinterventional surveillance program that included clinical evaluation by a consultant vascular surgeon and an aortoiliac computed tomography angiography (CT angiography) within the first postoperative month and annually during the first 5 years. After 5 years of follow-up, if no aneurysm growth was detected, a color duplex ultrasound was obtained annually. When clinically relevant in-stent stenosis was identified, patients were treated with balloon angioplasty, drug-eluting balloon therapy, or supplementary stent implantation. Detection of progressive aneurysm growth (>5 mm annually) or stenotic lesions in the iliac limb or branch vessels during monitoring prompted either endovascular intervention or open surgical correction.

### 2.3. Definitions and Outcome Measurement

Technical success criteria included the following: (1) successful IBD implantation in target iliac vessels, (2) preserved antegrade flow to internal iliac artery tributaries, (3) complete aneurysm exclusion without endoleak, (4) patent iliac segments (branch, CIA, and external iliac artery), and (5) no conversion to open surgery.

The primary outcome of this analysis was procedure and/or the aortoiliac aneurysm-related mortality during follow-up. Pelvic ischemia (bowel; spinal cord or nerve ischemia; erectile dysfunction; buttock or thigh claudication; and buttock necrosis) and failure to exclude the aortoiliac aneurysm were additional outcomes considered.

Reintervention was indicated for failure of aneurysm exclusion (aneurysm growth > 5 mm) or iliac limb or branch thrombosis during follow-up.

### 2.4. Statistical Analysis

Mean and standard deviation were used to describe continuous variables, median with range for follow-up periods, and absolute numbers with percentages for categorical variables. Baseline characteristics, clinical outcomes, and survival were analyzed. Analysis of the IBD-related outcomes, including branch occlusion or endoleak, and mortality was performed. Survival and reintervention rates were estimated using the Kaplan–Meier survival analysis on a per-patient basis. The χ^2^ and Fisher exact tests and the odds ratios (ORs) with 95% confidence intervals (CIs) were assessed for univariate analysis. Post hoc multivariable analysis explored the relationship between treatment strategy (bilateral simultaneous versus isolated CIA aneurysm repair with IBD) and the development of occlusions and endoleaks. In addition to the aforementioned variables, the regression model included surgical indication (indications included managing CIA aneurysms, type Ib endoleaks after EVAR, or AAA with a diameter at the common iliac artery bifurcation ≥ 20 mm), concomitant antiplatelet or anticoagulant therapy, and coexistence of hypogastric artery aneurysm. A significance level of 5% was used, with *p* < 0.05 considered statistically significant. All calculations were performed using SPSS statistical software (version 27; IBM Corporation, Somers, NY, USA).

## 3. Results

Between January 2014 and December 2024, a total of 81 patients were consecutively treated with the E-liac side-branched device. Most patients were male (80.2%), and their mean age was 71.2 ± 11.43 years (range 61–86 years). Hypertension (70.4%) and hyperlipidemia (50.6%) were the two most common risk factors. The most frequent American Society of Anesthesiologists classification was category III (61.7%) (Table 2). Two-thirds of the patients (66.7%) were treated preoperatively with aspirin, fifteen (18.5%) with clopidogrel, and twenty-one (25.9%) were anticoagulated.

Thirty-seven patients (45.7%) had aneurysmal disease of the iliac arteries, twenty-three patients (28.4%) had abdominal aortic aneurysm (AAA) with a diameter at the common iliac artery bifurcation ≥ 20 mm, and twenty-one patients (25.9%) had type Ib endoleak after EVAR. A total of 97 IIAs were revascularized; in 54 cases (66.7%), the endovascular technique was EVAR with unilateral IBDs, in 16 cases (19.8%), it was EVAR with bilateral IBDs, and in 11 patients (13.6%), it was unilateral isolated IBDs (Table 3). Of the 162 inguinal accesses performed, 87 (53.7%) were through open surgical exposure. Five patients (6.2%) presented coexisting hypogastric artery aneurysm. The total number of bridging stents implanted was 126 (1.6 per case); in 68 IBDs, a single stent-graft was used, and in 29 cases, two were used. E-ventus (Artivion/Jotec) was the most frequently used (67.5%); however, Gore Viabahn (14.3%), iVascular iCover (11.1%), and Atrium Advanta V12 (7.1%) were also employed. In 11 cases (13.6%), two different types of stent-grafts (balloon-expandable and auto-expandable) were required to achieve an adequate distal landing zone (Table 3).

Technical success was 100%. There were three in-hospital deaths (3.7%): one due to myocardial infarction, one due to pulmonary thromboembolism, and another due to colonic ischemia secondary to mesenteric and portal venous thrombosis (Table 4). One case of paraplegia was observed with complete recovery after cerebrospinal fluid drainage. No cases of stroke, nerve ischemia, or buttock necrosis were observed. Median hospital length of stay was 3.7 days (range 3–11 days). Ten patients (12.3%) presented wound complications requiring surgical revision.

**Figure 2:**
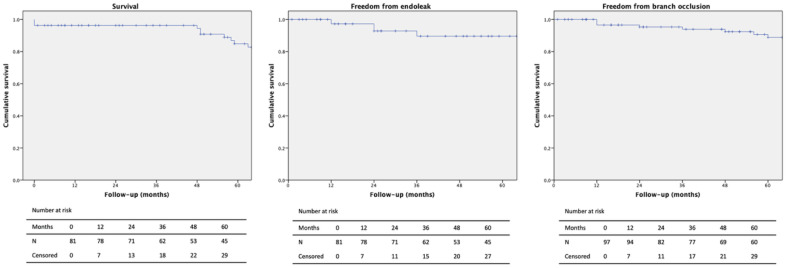
Kaplan–Meier curves of survival, freedom from endoleak, and freedom from occlusion. SE: Standard deviation.

The median patient follow-up time was 64.7 months (range 1–120 months). Conversion to open surgery was not necessary. During follow-up, 15 additional patients died, resulting in an overall mortality of 22.2% with no aneurysm-related deaths (Figure 2). The estimated survival at 1, 3, and 5 years was 96.3%, 88.9%, and 71.2%, respectively (Table 5). Eleven cases (13.6%) of myocardial infarction and one stroke (1.2%) occurred during follow-up. Buttock claudication was observed in five (6.2%) patients and nerve ischemia in one (1.2%). The patency of the iliac branch and bridging stent was 90.1%. One type I endoleak (1.2%) occurred after endovascular treatment of an isolated common iliac artery aneurysm, and three type II endoleaks (3.7%) were observed; however, none were associated with aneurysm sac enlargement during the radiological surveillance. Three type III endoleaks (3.7%) occurred due to disconnection of the iliac branch from the concomitant EVAR extension. Four reinterventions (4.9%) were performed during follow-up.

No statistically significant association between endoleak or occlusion and bilateral implantation was revealed (*p* = 0.716 and *p* = 0.185, respectively), or with isolated common iliac artery aneurysm treatment (*p* = 0.312 and *p* = 0.215, respectively) in our post hoc regression analysis.

## 4. Discussion

Preserving blood flow to the hypogastric arteries during EVAR is strongly recommended by current guidelines; therefore, IBDs have been increasingly used to maintain antegrade flow in the IIA during the last decade [9,10]. This study describes our eleven-year experience with the E-liac stent-graft system implantation for treating patients with aorto-iliac or isolated common iliac artery aneurysms. Despite the complexity of this patient population, our real-world experience demonstrates that effective management is feasible, yielding excellent clinical outcomes, durable patency, and infrequent secondary interventions.

Our 100% technical success is aligned with the results published in 2022 by Cao et al. [11]. This systematic review and meta-analysis of 2.736 patients treated with IBDs (six different types: IBE from Gore and Associates, ZBIS, ZHIS, and BB-IBD from Cook Medical, IBSG from Lifetech Scientific, and E-liac from Jotec) reported a pooled technical success rate of 98.0% (95% CI: 97.3–98.7%). The pELVIS registry [12], the largest European experience of more than 900 IBDs with a mean follow-up of 34 months, reported the onset of late type I endoleak and type III endoleak of 4.4% vs. 6.1% (*p* = 0.66) and 2.2% vs. 1.4% (*p* = 0.65) for isolated vs. non-isolated IBDs, respectively. Our results are better regarding the occurrence of type I endoleaks (1.2%) but slightly higher for type III endoleaks (3.7%); however, the mean follow-up of our series was longer (64.7 versus 34 months). In our series, we treated only 11 isolated iliac aneurysms with IBDs, with a single type I endoleak (9.1%) occurring during follow-up and no type II or III endoleaks. Other reviews estimated a pooled rate of endoleak of 11.9% (95% CI: 9.15–14.65%) [8]—12.68% (95% CI: 8.80–17.07%) [2] during follow-up. Turchino et al. [13] published a multicenter study of 96 patients evaluating the mid-term outcomes of the most commonly employed IBDs (Cook, Gore, and Jotec), showing similar branch instability after a 2-year follow-up with no statistical differences in terms of branch occlusion and branch-related endoleaks. Only the Jotec group, compared to the Gore group, showed a significant decrease in the maximum diameter of the aortic aneurysm sac from baseline. The 12.3% of wound-related complications requiring surgical revision may seem quite high; however, this can be explained by the fact that over the years, of the 162 inguinal accesses performed, 87 (53.7%) were through open surgical access, and the rest were percutaneous.

In 2024, D’Oria et al. [14] published the results of a European multicenter HYPROTECT Study, which evaluated the 2-year outcomes of the Gore Excluder IBD in 437 patients. They described a characteristic variable denominated iliac branch instability, defined as a composite cumulative endpoint of any hypogastric branch-related complication leading to aneurysm rupture, death, occlusion or stenosis/kink, disconnection, type 1 or 3 endoleak, or reintervention to maintain branch patency or to treat a separation or endoleak. After 2 years of follow-up, branch stability was 94% and 90% in patients with or without coexisting hypogastric artery aneurysms. In our study, this variable was 93.1% and 88.2% after the 2- and 5-year follow-ups, respectively.

None of the bridging covered stents available commercially are designed specifically to be used with IBDs. In our study, the most frequently used stent-graft (67.5%) was the E-ventus (Jotec, Hechingen, Germany). Bracale et al. [15] studied the effectiveness of this stent in the occurrence of type I–III endoleaks and device occlusion in patients undergoing IBD placement. They included 32 patients with a technical success of 91% (perioperative absence of proximal or distal type I endoleak or type III endoleak), and after a median follow-up of 15 months, they only reported one case of E-ventus occlusion, one type IB endoleak, and one type III endoleak, with none of them involving the branch device or the E-ventus graft. Our series presented three type III endoleaks (3.7%) due to disconnection of the iliac branch from the extension of the concomitant EVAR, without relation to the bridging stents. The HYPROTECT study [14] used bridging stent-grafts exclusively (auto-expandable 93.7% or balloon-expandable 6.3%) from Gore and reported that 5.8% of endoleaks were related to the IBD. Barnes et al. published results in 2023 on the combined use of the Gore Excluder IBD and the Gore Viabahn VBX balloon-expandable stent in 30 patients with no reinterventions after a 1-month follow-up [16]. A systematic review and meta-analysis comparing balloon-expandable versus self-expandable stent grafts used with IBDs has been published this year [17]. It included 684 patients treated with 711 IBDs, but they only considered three studies using Gore IBD in combination with Viabahn or Viabahn VBX, and one study using Cook IBD in combination with the Fluency or Advanta V12 stents-grafts. They found that the composite of all endoleaks was significantly less common in patients treated with self-expanding compared to balloon-expandable stent-grafts (OR 5.53; 95% CI 1.15 to 26.63; *p* = 0.03). No differences were found with respect to type Ib, type Ic, type III endoleaks, buttock claudication, internal iliac artery occlusion, and IBD-related reinterventions.

The largest prospective study on iliac side branch devices was published this year, the PLIANT II Study [18]. This European multicenter study included 295 patients treated with the E-liac stent-graft system for the treatment of iliac aneurysms. Two hundred thirty-six patients were also treated for a concomitant AAA. At the 12-month follow-up, freedom from occlusion was 94.5% for the external iliac artery and 94.7% for the internal iliac artery. The survival rate was 96.7%, a similar result to our series (96.3%). These results make the E-liac stent-graft system comparable with other IBDs. Mylonas et al. [19] compared the evolution of 84 consecutive patients (44 treated with the E-liac and 40 treated with the Zenith ZBIS) with no statistically significant differences in freedom from reintervention or freedom from occlusion after more than 1-year follow-up (86.0% vs. 87.2%, *p* = 0.563, and 95.3% vs. 89.7%, *p* = 0.317, respectively). The E-liac stent-graft system has even demonstrated appropriate results in combination with the Endurant II or IIs (Medtronic^®^) for the treatment of aortoiliac aneurysms, with an IBD-related reintervention rate of 2.6% [20].

In our series, the survival after one-year follow-up was 96.3%; nevertheless, during the median follow-up of more than 5 years, the overall mortality was 22.2% with no aneurysm-related deaths. The published survival after one-year follow-up related to E-liac implantation has been between 95.2% and 98.5% [9,18,20]. No publication reported aneurysm-related mortality during follow-up, except for Yazar et al. [20]. They included 63 patients treated with the E-liac device in their study, and after a median follow-up time of 38 months, 74.6% of patients were still alive, with only one aneurysm-related death. In a study comparing 40 patients treated with ZBIS and 44 with E-liac, the cumulative mortality after a median follow-up period of 37 months was 5% and 2.3%, respectively [19].

The E-liac device has also demonstrated its utility for the endovascular exclusion of hypogastric artery aneurysms. Distal extension of the repair beyond the hypogastric artery bifurcation is normally performed into the anterior or posterior divisional branches, while using plugs or coils to exclude the other. Dueppers et al. [21] reported 18 IBDs implanted in 14 male patients with internal iliac aneurysms. The technical success rate was 100% and after a mean follow-up of 19 months, the related mortality was 0%. Nevertheless, two hybrid and four endovascular reinterventions were required, resulting in a 42.9% reintervention rate, requiring advanced endovascular experience with special techniques for prevention of endoleaks, especially type II endoleaks. In our series, five patients presented concomitant hypogastric artery aneurysms, and only one of them presented asymptomatic branch occlusion during follow-up. Other devices, such as the Zenith ZBIS IBD, have reported up to 37% of iliac reinterventions after a mean time of 3-month follow-up [22].

Our series included 11 patients (13.6%) with solitary iliac branch endoprosthesis placement for iliac artery aneurysms. The theoretical advantage of this endovascular strategy is to decrease the risk of occluding the lumbar arteries or inferior mesenteric artery, reducing the risk for pelvic ischemia and reducing procedural time and costs [1]. During follow-up, we observed one type Ia endoleak in this group of patients. Oussoren et al. [23] in 2021 published a multicenter retrospective study of 51 patients with 54 isolated iliac artery aneurysms. In their series, after a 2-year follow-up, only six endoleaks (one type Ia, two type Ib, two type II, and one type III) were observed in five patients.

Manifestations of pelvic ischemia after IBD implantation related to sexual function remain relatively scarce in the literature [24]. This year, Xu et al. [25] published their results for the treatment of patients with aortoiliac aneurysms using IBDs. They included 76 patients, and to accurately assess sexual dysfunction, the male patients were asked to complete the International Index of Erectile Function-5 (IIEF-5) during both preoperative and final postoperative follow-up visits. Two patients (5.3%) in the group of patients with bilateral iliac artery aneurysms were treated with bilateral implantation of IBDs, and twelve patients (31.6%) in the group of patients with bilateral aneurysms underwent coil or plug occlusion of one IIA, while the other was preserved using an IBD. The group treated with IIA occlusion presented a statistically significant decrease in the IIEF-5 classification score (*p* < 0.05).

This work has inherent limitations. Being a single-center study, it carries risks of selection bias and data variability that may compromise external validity. The observational, non-comparative design without randomization potentially influenced the findings. The absence of independent outcome adjudication and extended follow-up further limits the ability to establish firm conclusions. Iliac tortuosity has been described as a risk factor for overall complications and reinterventions, particularly for IBD-related endoleaks [21]; nevertheless, it was not studied in our series because our endpoints were clinical and device-related, not anatomical. Despite collecting clinical data such as buttock claudication or nerve ischemia, the inclusion of quality of life scales or erectile function assessment could be useful in future studies. However, this cohort is the largest single-center study published in the literature and the only one with a mean follow-up longer than five years with the implantation of the E-liac stent-graft system for the treatment of aorto-iliac or isolated common iliac artery aneurysms, and provides valuable real-world experience.

## 5. Conclusions

This 11-year experience demonstrates that the endovascular approach for the treatment of aortoiliac or isolated common iliac aneurysms is safe and durable. The rates of major complications, including occlusions and endoleaks, are acceptable. The E-liac branched stent-graft system showed encouraging mid- and long-term results, with high technical success and low reintervention rates, confirming the efficacy of the device.

## Figures and Tables

**Table 1:** Anatomical complexity and vascular morphology of aneurysms included in the study. Data are presented as mean ± standard deviation. CIA: common iliac artery; IIA: internal iliac artery; EIA: external iliac artery; AAA: abdominal aortic aneurysm.

CIA diameter (mm)	34.3 ± 9.3
IIA diameter (mm)	7.9 ± 8.2
EIA diameter (mm)	11.2 ± 5.4
Angle between EIA/IAA (°)	57.2 ± 21.3
Aortic neck diameter (mm)	27.1 ± 5.3
Aortic neck length (mm)	22.1 ± 7.6
AAA (mm)	57.9 ± 8.8
Thrombus free lumen above iliac bifurcation (mm)	21.3 ± 7.5

**Table 2:** Baseline characteristics and preoperative details of patients undergoing treatment of aortoiliac aneurysm with the E-liac stent-graft system. Data are presented as mean ± standard deviation or *n* (%). COPD: chronic obstructive pulmonary disease; CHF: congestive heart failure; CAD: coronary artery disease; PAD: peripheral artery disease; AAA: abdominal aortic aneurysm; EVAR: endovascular aneurysm repair.

	*n* = 81	%
Age (years mean, SD)	71.2 ± 11.43	range (61–86)
Gender (male)	65	80.2%
Hypertension	57	70.4%
Hyperlipidemia	41	50.6%
Diabetes mellitus	35	43.2%
History of smoking	55	67.9%
PAD	27	33.3%
CAD	18	22.2%
Renal insufficiency	16	19.8%
COPD	11	13.6%
CHF	9	11.1%
Cerebrovascular disease	8	9.9%
History of cancer	8	9.9%
Active cancer	3	3.7%
ASA classification		
II	17	21.0%
III	50	61.7%
IV	14	17.3%
Aspirin	54	66.7%
Clopidogrel	15	18.5%
Anticoagulation	21	25.9%
Indication		
Iliac aneurysm	37	45.7%
AAA with diameter at common iliac artery bifurcation > 20 mm	23	28.4%
Type Ib endoleak after EVAR	21	25.9%

**Table 3:** Details of the endovascular procedures for treatment of aortoiliac aneurysms.

	*n* = 81	%
EVAR + unilateral IBD	54	66.7%
EVAR + bilateral IBD	16	19.8%
Previous EVAR	21	25.9%
Unilateral isolated IBD	11	13.6%
Bilateral isolated IBD	0	0.0%
Coexisting hypogastric artery aneurysm	5	6.2%
Bridging stent-graft per vessel		1.6%
Total bridging stent-grafts	126	
E-ventus	85	67.5%
Gore Viabahn	18	14.3%
iCover	14	11.1%
Advanta V12	9	7.1%
Combined cases	11	13.6%

**Table 4:** Procedural results and in-hospital follow-up.

	*n* = 81	%
Primary technique success	81	100.0
Intraoperative endoleaks	0	0.0%
ICU stay	1.3 ± 3.1	range 1–11
Hospital stay	3.7 ± 2.9	range 3–11
Mortality	3	3.7%
Myocardial infarction	1	1.2%
Paraplegia	1	1.2%
Colonic ischemia	1	1.2%
Stroke	0	0.0%
Nerve ischemia	0	0.0%
Buttock necrosis	0	0.0%
Wound complications needing revision	10	12.3%

**Table 5:** Mid-term follow-up.

	*n* = 81	%
Follow-up (months)	64.7 (1–132)	
Mortality	18	22.2%
Stroke	2	2.5%
Myocardial infarction	12	14.8%
Paraplegia	1	1.2%
Nerve ischemia	1	1.2%
Colonic ischemia	1	1.2%
Buttock claudication	5	6.2%
Conversion to open repair	0	0.0%
Re-interventions	4	4.9%
Endoleaks	7	8.6%
Type I	1	1.2%
Type II	3	3.7%
Type III	3	3.7%
Patency of iliac branch and bridging-stent	88	90.1%

## Data Availability

The data underlying this article will be shared upon reasonable request to the corresponding author.

## Supplementary Materials

The following supporting information can be downloaded at: https://www.mdpi.com/article/10.3390/jcm14228203/s1, Table S1: STROBE Statement—Checklist of items that should be included in reports of cohort studies.
